# Evaluating the Quality of Guidelines Using the AGREE II Tool by a Large Language Model vs Human Appraisers

**DOI:** 10.1001/jamanetworkopen.2025.12621

**Published:** 2025-05-28

**Authors:** Bingyi Wang, Xufei Luo, Jie Zhang, Qianling Shi, Honghao Lai, Hui Liu, Yuanyuan Yao, Long Ge, Jiayi Liu, Haodong Li, Yanfang Ma, Lu Zhang, Zhaoxiang Bian, Ivan D. Florez, Yaolong Chen, Janne Estill

**Affiliations:** 1 Evidence-Based Medicine Center, School of Basic Medical Sciences, Lanzhou University, Lanzhou, Gansu, China; 2 Research Unit of Evidence-Based Evaluation and Guidelines, Chinese Academy of Medical Sciences (2021RU017), School of Basic Medical Sciences, Lanzhou University, Lanzhou, Gansu, China; 3 World Health Organization Collaboration Center for Guideline Implementation and Knowledge Translation, Lanzhou, Gansu, China; 4 Institute of Health Data Science, Lanzhou University, Lanzhou, Gansu, China; 5 Key Laboratory of Evidence Based Medicine of Gansu Province, Lanzhou University, Lanzhou, Gansu, China; 6 The First School of Clinical Medicine, Lanzhou University, Lanzhou, Gansu, China; 7 Department of Health Policy and Management, School of Public Health, Lanzhou University, Lanzhou, Gansu, China; 8 School of Public Health, Lanzhou University, Lanzhou, Gansu, China; 9 Vincent V.C. Woo Chinese Medicine Clinical Research Institute, School of Chinese Medicine, Hong Kong Baptist University, Hong Kong, China; 10 Chinese EQUATOR Centre, Hong Kong, China; 11 Department of Computer Science, Hong Kong Baptist University, Hong Kong, China; 12 Department of Pediatrics, University of Antioquia, Medellin, Colombia; 13 Pediatric Intensive Care Unit, Clínica Las Américas-AUNA, Medellin, Colombia; 14 School of Rehabilitation Science, McMaster University, Hamilton, Canada; 15 Institute of Global Health, University of Geneva, Geneva, Switzerland

## Abstract

This quality improvement study examines the efficacy of a large language model to evaluate guidelines for therapeutic drug monitoring compared with human apparaisers.

## Introduction

Clinical practice guidelines underpin evidence-based care, yet methodological inconsistencies necessitate robust quality assessment. While the AGREE II (Advancing Guideline Development, Reporting and Evaluation in Health Care II) persists as the most widely adopted framework for guideline appraisal, its application requires 2 to 4 trained assessors investing 1.5 hours each per guideline, posing implementation challenges.^1,2^ Large language models (LLMs) have potential to automatically extract information from guidelines and assess the quality of the studies.^3,4^ However, to the best of our knowledge, no study has utilized LLMs to assess the methodological quality of guidelines with the AGREE II instrument.

## Methods

This study adhered to the Guidelines for Reporting Reliability and Agreement Studies (GRRAS).^5^ We selected 28 guidelines on therapeutic drug monitoring, published between 1995 and 2018, previously evaluated by human appraisers using AGREE II (eAppendix 1 in Supplement 1).^6^ GPT-4o (Open AI) evaluated these guidelines 4 times using prompts designed by our group (eFigure 1 and eTable in Supplement 1). We compared the domain-specific AGREE II assessment results of the LLM with those of human appraisers using the intraclass correlation coefficient (ICC) and Bland-Altman plots. Internal consistency and item-level consistency were also analyzed (eFigure 2 in Supplement 1). Evaluation time was recorded. See eMethods in Supplement 1 for additional details.

## Results

**Figure 1.**
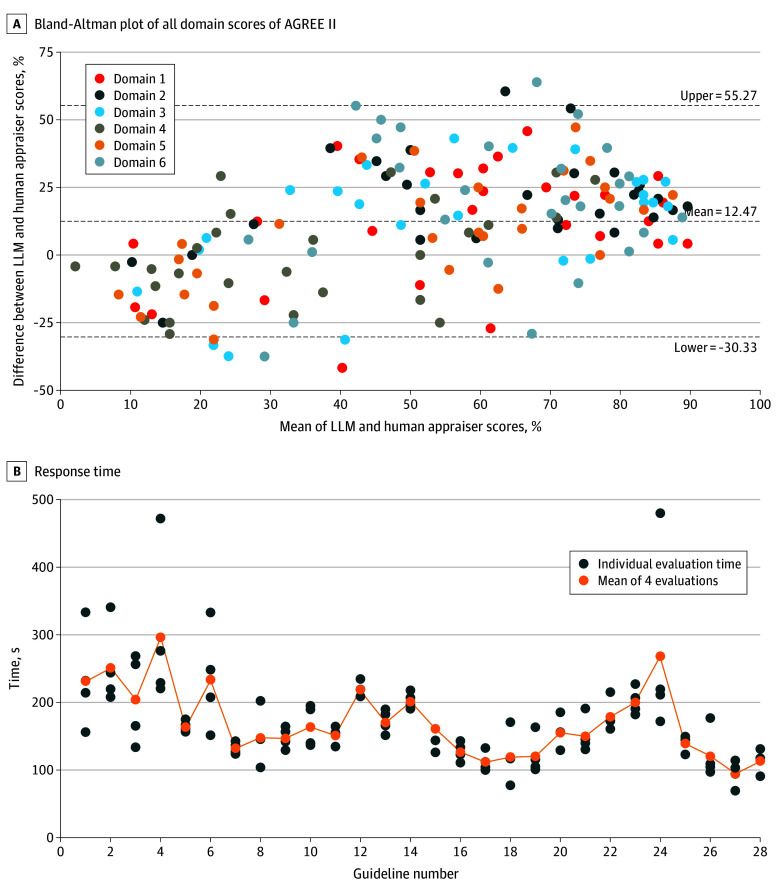
Large Language Model (LLM) Performance The internal consistency between the 4 evaluations was high (intraclass correlation coefficient, 0.791; consistency index, 0.863).

**Figure 2.**
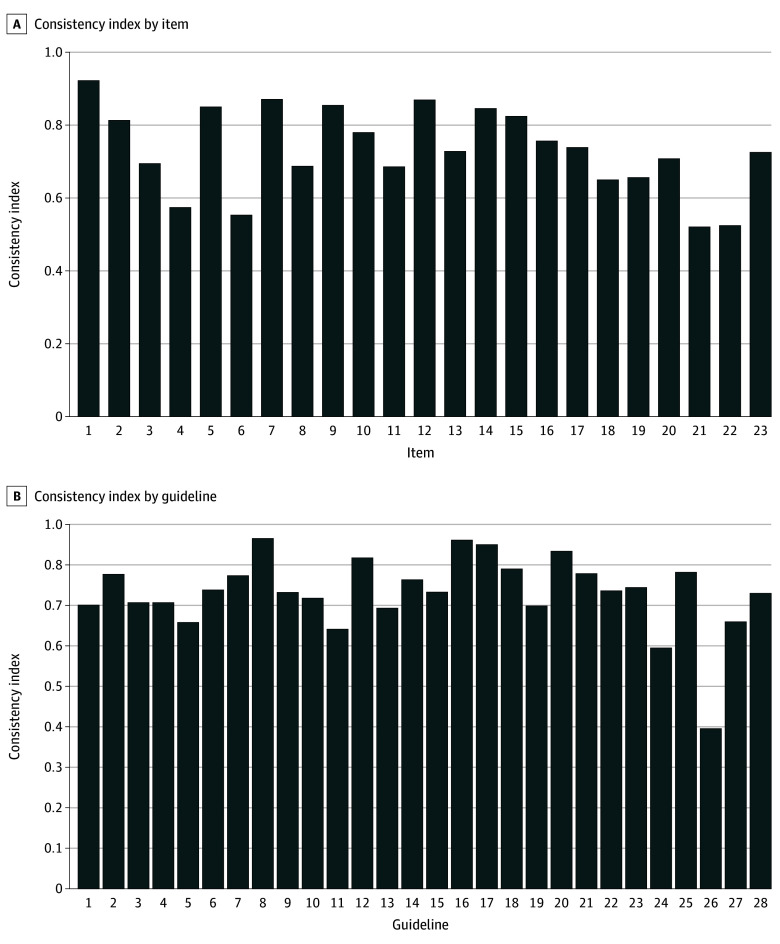
Consistency Indices of Each Item and Guideline See eAppendix in Supplement 1 for full details on the guidelines.

The overall consistency of the 4 evaluations by an LLM compared with human appraisers was substantial (ICC, 0.753; 95% CI, 0.532-0.854). Only 4 guidelines had ICC below 0.4. A comparison of the scores for each domain between the LLM and human appraisers using a Bland-Altman plot revealed 81.5% of domain scores within the acceptable range (33.3%) of human ratings. The LLM generally gave higher scores than humans (mean difference, 12.5%; 95% limits of agreement [LoA], −30.6% to 55.5%) (Figure 1A). Domain 4 (clarity of presentation) demonstrated the best evaluation performance, with a mean difference of −0.2% (95% LoA, −35.2% to 35.0%), with notable overestimation in domain 2 (stakeholder involvement: mean difference, 22.3%; 95% LoA, −13.2% to 53.8%). The mean (SD) evaluation time per guideline was 171 (SD) seconds (Figure 1B). The mean consistency (SD) index across all guidelines calculated item-wise was 0.732 (0.177), with only 2 guidelines having a consistency index below 0.6. Items 4, 6, 21, and 22 had the lowest item-specific consistency (index below 0.6) (Figure 2).

## Discussion

In this study, the LLM could evaluate a guideline within approximately 3 minutes. Low consistency in certain guidelines likely stems from unconventional formats or ambiguous content. The LLM scored slightly higher than humans on high-quality guidelines, likely because it can make reasonable inferences based on the existing information. Humans’ scores exceeded the LLM’s for lower-quality guidelines, possibly because the experience of human experts or subjective judgment in ambiguous cases.

This study has several limitations. The LLM we used overlooks font-based cues (eg, bold text for key recommendations), we relied on published human scores, the sample of guidelines was small of the same type, and there is a risk of inherent biases. Another limitation was that supplemental materials referenced in some guidelines were not included in the uploaded files. While this had minimal impact in most cases, evidence tables were missing in 1 instance. Nevertheless, the LLM’s evaluations aligned with human scores, hinting at possible link-following capabilities.

An LLM’s ability to quickly exclude low-quality guidelines from consideration could streamline clinical decision-making, reducing reliance on suboptimal guidelines. Future work should study more diverse samples of guidelines, explore other LLMs, and enhance prompts to tackle complex items, unlocking LLMs’ full potential in evidence-based practice.

## Notes

Supplement 1.
eMethods.
**eFigure 1.** Flowchart of the Study Process **eTable.** S-Prompting **eFigure 2.** Calculation of the Consistency Index: Pairwise Comparisons Between GPT-4o Queries and Human Appraisers **eAppendix.** References of the 28 Guidelines Included

Supplement 2.
Data Sharing Statement
