# Supplementary material for: Evaluating the Quality of Guidelines Using the AGREE II Tool by a Large Language Model vs Human Appraisers
Source: JAMA Netw Open. 2025 May 28;8(5):e2512621. doi: 10.1001/jamanetworkopen.2025.12621 (PMC12120650; doi:10.1001/jamanetworkopen.2025.12621)
Supplement: Supplement 1. — eMethods. eFigure 1. Flowchart of the Study Process eTable. S-Prompting eFigure 2. Calculation of the Consistency Index: Pairwise Comparisons Between GPT-4o Queries and Human Appraisers eAppendix. References of the 28 Guidelines Included [file jamanetwopen-e2512621-s001.pdf]

Supplemental Online Content

Wang B, Luo X, Zhang J, et al. Evaluating the quality of guidelines using the AGREE II tool by GPT-4o vs human appraisers. *JAMA Netw Open.* 2025;8(5):e2512621. doi:10.1001/jamanetworkopen.2025.12621

eMethods

eFigure 1. Flowchart of the Study Process

eTable. S-Prompting

eFigure 2. Calculation of the Consistency Index: Pairwise Comparisons Between GPT-4o Queries and Human Appraisers

eAppendix. References of the 28 Guidelines Included

This supplemental material has been provided by the authors to give readers additional information about their work.

© 2025 Wang B et al. *JAMA Network Open*.

eMethods

We used the web version of GPT-4o to assess the quality of selected guidelines, extracted the scores given by human appraisers on the same guidelines from published literature, and compared the results. **eFigure 1** illustrates the process.

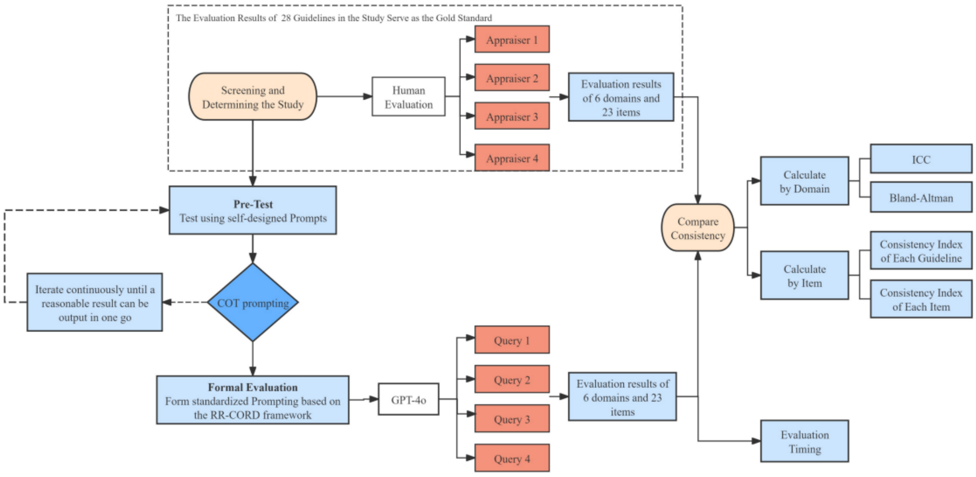

eFigure 1. *Flowchart of the study process. Query 1, Query 2, Query 3 and Query 4 are the four evaluations conducted using GPT-4o; COT: Chain of Thought; ICC: Intraclass Correlation Coefficient; RR-CORD is a framework for guiding tasks by defining the Role, Response Rules, Context, Objective, Response Format, and Descriptions, including examples.*

1.1 Selection of the sample of guidelines

We selected a study by Min Zeng et al., published in the Journal of Clinical Epidemiology, as the source of human guideline evaluations[1]. The study strictly adhered to the user manual[2] in employing the AGREE II instrument. Four appraisers were involved in the assessment process, all of whom had undergone specialized training and pro-seminar on the application of AGREE II. The appraisers demonstrated good inter-rater agreement (ICC values ranging from 0.700 to 0.880). Additionally, the study provided detailed evaluation results for each item, rather than just for each domain, and all original guidelines were available. The selected study assessed 28 guidelines, and the evaluation results were chosen as the human benchmark for comparison.

1.2 AGREE II Instrument

The AGREE II Instrument, developed by the AGREE Next Steps Consortium, consists of six domains with a total of 23 items: Domain 1 (Scope and purpose, items 1-3); Domain 2 (Stakeholder involvement, items 4-6); Domain 3 (Rigor of development, items 7-15); Domain 4 (Clarity of presentation, items 16-19); Domain 5 (Applicability, items 20-21); and Domain 6 (Editorial independence, items 22-23). A minimum of two appraisers, preferably four, independently evaluate the quality of the guidelines or consensus statements. Each item is rated on a 7-point scale, 1 indicating "strongly disagree" and 7 indicating "strongly agree". Domain scores are calculated using the formula:

© 2025 Wang B et al. *JAMA Network Open*.

$$\text{Domain score} = \frac{\text{Obtained score} - \text{Minimum possible score}}{\text{Maximum possible score} - \text{Minimum possible score}} \times 100\%$$

1.3 Optimization and testing of the prompt

We used the Chain of thought (COT) strategy in combination with the basic elements of prompt generation frameworks to construct the prompts to be used in this study. The COT strategy breaks tasks into different steps to execute the required reasoning process. Previous research has shown that COT improves the performance of LLMs in reasoning-based tasks through few-shot learning[3]. By optimizing and managing the selection of COT examples, the performance of pretrained models like GPT-4o can be further enhanced[4]. Some studies have also applied this strategy to guideline development, showing promising results[5].

All evaluations were conducted using the default configuration of GPT-4o (temperature parameter = 1.0, no adjustments to top-p or other hyperparameters) via the OpenAI web interface without additional API customization. To ensure transparency in reporting, we followed the recommendations by Perlis and Fihn[6] for documenting the use of large language models in clinical research. Detailed descriptions of the prompt engineering process, including the RR-CORD framework and the standardized prompts (S-prompt), are provided in eTable 1.

1.3.1 Pre-test

Before starting the formal evaluation, we conducted a pilot test using another study that evaluated the quality of 15 guidelines to test and iteratively refine the prompt[7]. We first used COT prompting strategy to instruct GPT-4o to complete the task step by step according to a specific reasoning pathway, and revised the prompts iteratively. To avoid generating seemingly convincing but factually incorrect text (commonly referred to as "hallucinations")[8], we instructed the model to present the specific content from the original text of the guideline that was used for the evaluation. The process was continued until GPT-4o gave the required output, i.e. an evaluation score and the original text from the guideline that the justification was based on, rather than some other content such as explaining how AGREE II works or asking the user to evaluate the guidelines themselves according to the standards.

Finally, we formed a custom prompting framework[9] We named this framework as RR-CORD, standing for R (Role; define the role or perspective from which the task should be approached); R (Response Rules; outline the overarching principles or guidelines for answering the questions); C (Context; provide background information on the task); O (Objective; define the specific task you want the language model to perform); R (Response Format; specify the desired format of the response); D (Descriptions; add detailed descriptions to the questions, including examples).

The "Descriptions" section of the RR-CORD framework was standardized, ultimately leading to the creation of a "standardized prompt", referred to as S-prompting (**eTable 1**). We established formal scoring criteria for each item[10-11]. This involved providing specific guidance for each score on the 7-point AGREE II evaluation scale. This guidance was developed through consensus among the members of the research team, who have extensive experience in guideline evaluation.

The final prompts were structured using the RR-CORD framework (**eTable 1**), which included explicit instructions for scoring criteria and examples to minimize ambiguity.

1.3.2 Formal evaluation

The formal evaluation, where the model was employed to evaluate the quality of the full sample of 28 guidelines using the S-prompt described in the previous section, was conducted on June 7, 2024 using the latest available web-based version of GPT-4o.

© 2025 Wang B et al. *JAMA Network Open*.

1.4 Data collection and processing

To ensure the reliability of the results, each guideline was evaluated multiple times, corresponding to the number of appraisers in the original study, using the same prompt. GPT-4o was therefore requested to perform four evaluations, simulating four different appraisers. The evaluation process was conducted via the web chat interface, with each guideline's PDF file uploaded to the chat window, and the prompt used to trigger responses. Each evaluation was carried out in a separate conversation to avoid the influence of the previous evaluations on the answers. Due to the excessive length of the S-prompts, which may negatively affect the performance of GPT-4o, we divided the S-prompt into three parts based on the AGREE II domains during the evaluation process. These parts were then input sequentially in a single dialogue box (domains 1 and 2: items 1-6; domain 3: items 7-14, and domains 4, 5 and 6: items 15-23) to mitigate the impact of prompt length on the model's performance. Evaluations interrupted by network issues and those that did not follow the prompt instructions and provided answers without scores were excluded and immediately redone to maintain data integrity.

1.5 Outcome measurement

1.5.1 Domain-level consistency for each guideline

We used the Intraclass Correlation Coefficient (ICC) to compare the LLMs' domain-specific AGREE II assessment results with those of the human appraisers from the study we selected, calculating ICC values for all guidelines. ICC values range from 0 to 1, with benchmarks defined according to previous studies[12-17]: ICC up to 0.20 indicates slight consistency, >0.20 to 0.40 fair consistency, >0.40 to 0.60 moderate consistency, >0.60 to 0.80 substantial consistency, and >0.80 almost perfect consistency.

A Bland-Altman plot (also known as a difference plot or limits of agreement plot) was used to compare the differences between GPT-4o and human appraisers in their AGREE II domain scores. Since we assessed six domains of AGREE II for each domain, the number of difference points is six times the number of included guidelines. The Bland-Altman plot shows the mean difference (bias) between the two evaluations (GPT-4o and human) and the 95% limits of agreement (95% LoA; ±1.96 standard deviations)[18]. Considering that appraisers often face difficulties assigning mid-range scores (3-5) on the AGREE II scale[19], we established a scoring standard in our "Descriptions" prompt that allows for a maximum fluctuation of two points per item. Consequently, a score difference of up to two points between GPT-4o and human appraisers for each item is considered acceptable. The acceptable deviation for AGREE II domain evaluation scores was thus set at 33.3%. When all differences were within ±33.3%, consistency was deemed to be good.

1.5.2 Internal consistency of GPT-4o

Internal consistency across the four assessments conducted by GPT-4o was evaluated using two metrics: the ICC and a consistency index. The consistency index for each guideline was calculated by comparing the differences in scores between all pairs taken from the four queries. The number of times the absolute difference in scores was ≤2 points was divided by the total number of comparisons (6 pairwise comparisons per guideline multiplied by 23 items) to calculate the consistency index.

1.5.3 Item-level consistency index for each guideline

We compared the four GPT-4o assessments pairwise with the assessments of the four human appraisers, resulting in a total of 16 GPT-human comparisons per guideline (**eFigure 2**). For each comparison, we calculated the number of items for which the absolute difference between the scores

was no more than 2. We then added these numbers together over all 16 comparisons and defined the consistency index as the sum divided by 16*23. We also calculated the consistency score for each item over all guidelines in a similar manner.

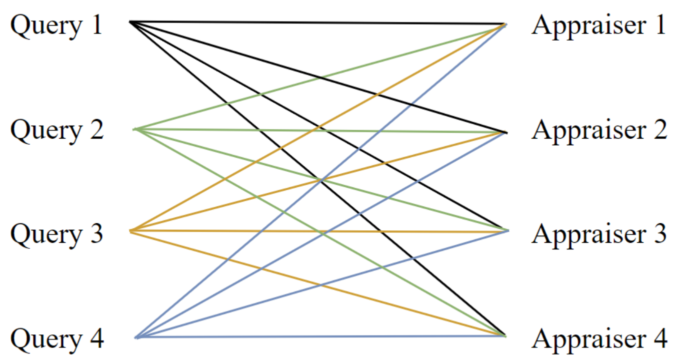

eFigure2. *Calculation of the consistency index: pairwise comparisons between GPT-4o queries and human appraisers*

Method for calculating the consistency for each guideline:

Formula for score difference calculation:

For each item *j* in the guideline, the score difference $\Delta_{ij,\ kj}$ between a human appraiser *i* and GPT-4o query *k* was calculated as follows:

$$\Delta_{ij,\ kj}=|\mathrm{Score}_{ij}-\mathrm{Score}_{kj}|$$

Determination of consistency:

If $\Delta_{ij,\ kj}\leq 2$, it is considered that appraiser *i* and query *k* have consistent scores for item *j*, and it is denoted as $\delta_{ij,\ kj}=0$; otherwise, $\delta_{ij,\ kj}=1$.

Formula for guideline consistency index calculation:

For each guideline *n*, we calculated the proportion of consistent scores among all possible combinations:

$$\mathrm{Consistency}_n=\frac{\sum\delta_{ij,\ kj}=0}{16\times 23}$$

where the denominator 16×23 represents the total number of pairwise comparisons for that guideline (16 is the number of pairwise combinations, and 23 is the number of items).

Calculation of consistency for a single item:

For each item *j*, we counted the total number of times the score difference was up to two (recorded as 0) across all guidelines, and then calculated the consistency index for that item:

© 2025 Wang B et al. *JAMA Network Open*.

$$\text{Item consistency}_j = \frac{\sum \delta_{ij,\ kj} = 0}{16 \times N}$$

where *N* is the total number of evaluated guidelines, and the denominator 16×*N* represents the total number of pairwise comparisons across all guidelines.

1.5.4 Time needed for evaluation

To minimize discrepancies between different timekeepers, all timing was recorded by a single individual. The time needed for GPT-4o to evaluate each guideline was measured by starting the timer simultaneously with the click of the upload button after inputting the prompt into the dialog box, and stopping it immediately when GPT-4o completed its final output.

1.6. Statistical analysis

For normally distributed count data, the results were expressed as the mean ± standard deviation. For data that did not follow a normal distribution, the results were expressed as the median with interquartile range (IQR). The ICC values and Bland-Altman plots were calculated and generated using IBM SPSS Statistics 27 and R version 2024.12.1+563.

1.7 eReferences

[1] Zeng M, Yi Q, Zeng L, et al. Quality of therapeutic drug monitoring guidelines is suboptimal: an evaluation using the Appraisal of Guidelines for Research and Evaluation II instrument. J Clin Epidemiol. 2020;120:47-58.

[2] AGREE Next Steps Consortium. (2017). The AGREE II Instrument [User's Manual and 23-item Instrument]. Retrieved June 20, 2024, from https://www.agreetrust.org/wp-content/uploads/2017/12/AGREE-II-Users-Manual-and-23-item-Instrument-2009-Update-2017.pdf

[3] Wei, Jason, et al. Chain of thought prompting elicits reasoning in large language models. arXiv preprint arXiv: 2201.11903 (2022).

[4] Huang, X., Zhang, L.L., Cheng, K., et al. Fewer is More: Boosting LLM Reasoning with Reinforced Context Pruning. arXiv (2023).

[5] Wang L, Chen X, Deng X, et al. Prompt engineering in consistency and reliability with the evidence-based guideline for LLMs. NPJ Digit Med. 2024;7(1):41. Published 2024 Feb 20.

[6] Perlis RH, Fihn SD. Evaluating the Application of Large Language Models in Clinical Research Contexts. JAMA Netw Open. 2023;6(10):e2335924. Published 2023 Oct 2.

[7] Montero-Odasso MM, Kamkar N, Pieruccini-Faria F, et al. Evaluation of Clinical Practice Guidelines on Fall Prevention and Management for Older Adults: A Systematic Review [published correction appears in JAMA Netw Open. 2023 Aug 1;6(8):e2332257.

[8] Truhn D, Weber CD, Braun BJ, et al. A pilot study on the efficacy of GPT-4 in providing orthopedic treatment recommendations from MRI reports [published correction appears in Sci Rep. 2024 Mar 5;14(1):5431.

[9] Liu P, Yuan W, Fu J, et al. Pre-train, prompt, and predict: a systematic survey of prompting methods in natural language processing. arXiv. Preprint posted online July 28, 2021. arXiv:2107.13586. Accessed June 28, 2024.

[10] MacDermid JC, Brooks D, Solway S, et al. Reliability and validity of the AGREE instrument used by physical therapists in assessment of clinical practice guidelines. BMC Health Serv Res. 2005;5:18.

© 2025 Wang B et al. *JAMA Network Open*.

[11] Wimpenny P, van Zelm R. Appraising and comparing pressure ulcer guidelines. Worldview Evid-Based Nurs. 2007;4(1):40–50.
[12] Shrout PE. Measurement Reliability and Agreement in Psychiatry. Stat Methods Med Res (1998) 7(3):301–17.
[13] Zhang Y, Li ZH, Li SW, et al. The quality of guidelines on the pancreatic perioperative enhanced recovery after surgery: a systematic quality appraisal using AGREE II instrument. Support Care Cancer. 2024;32(7):456.
[14] Salmon MK, Fritz CG, Barrette LX, et al. Quality appraisal of clinical practice guidelines for the evaluation and management of primary hyperparathyroidism. Endocrine. 2024;85(2):864-872.
[15] Armstrong JJ. Rodrigues IB, Wasiuta T, et al. Quality assessment of osteoporosis clinical practice guidelines for physical activity and safe movement: an AGREE II appraisal. Arch Osteoporos. 2016;11:6.
[16] Messina C, Bignotti B, Bazzocchi A, et al. A critical appraisal of the quality of adult dual-energy X-ray absorptiometry guidelines in osteoporosis using the AGREE II tool: An Euro AIM initiative. Insights Imaging. 2017;8:311–317.
[17] Landis J.R., Koch G.G. The measurement of observer agreement for categorical data. Biometrics. 1977;33(1):159–174.
[18] Giavarina D. Understanding Bland Altman analysis. Biochem Med (Zagreb). 2015;25(2):141-151.
[19] Grimmer K, Dizon JM, Milanese S, et al. Efficient clinical evaluation of guideline quality: development and testing of a new tool. BMC Med Res Methodol. 2014;14:63.

© 2025 Wang B et al. *JAMA Network Open*.

eTable 1. *S-prompting*

**S-prompting**<br>【First input】(Simultaneously uploaded the guideline file)<br>**Your role**:<br>Suppose you are a clinical practice guideline developer and an evaluator of the methodological quality of clinical practice guidelines.<br><br>**Your task**:<br>Your task is to conduct a thorough critical appraisal of the submitted clinical practice guideline.. Your insights will contribute to an in-depth understanding of the guideline's quality and whether its development methods are rigorous and transparent.. Please utilizing your expertise, respond to the questions listed in the following **Questions part** based on the following **Response rules** and **Question descriptions**. **Question descriptions** add additional detailed descriptions to the questions listed in **Questions part**. The **Question descriptions** consists of three parts: Description, Locating Information, and How to Rate. These parts provide detailed descriptions for the questions listed in the **Questions part**.<br><br>**Response rules**:<br>1. All questions are rated on the following 7-point scale:<br>Score of 1 (Strongly Disagree): A score of 1 should be given when there is no information that is relevant to the question, if the concept is very poorly reported, or if the authors state explicitly that criteria were not met.<br>Score of 7 (Strongly Agree): A score of 7 should be given if the quality of reporting is exceptional and meets all the criteria and conditions required.<br>Scores between 2 and 6: A score between 2 and 6 is assigned when the reporting of the question does not meet the full criteria or considerations. A score is assigned depending on the completeness and quality of reporting. Scores increase as more criteria are met and considerations addressed.

© 2025 Wang B et al. *JAMA Network Open*.

2. For questions to which you rated with ‘1’, please give your detailed explanation. For questions to which you rated with ‘2, 3, 4, 5, 6, or 7’, please list the original and unmodified content from the uploaded article in an ascending order, formatted as 1, 2, 3, etc..

3. Each response should be informed and supported by the question description, ensuring a clear connection between the question asked and the description given.

Response Example:

Question 1):

Score: 1/2/3/4/5/6/7

Original and unmodified content from the uploaded article:

1. xxxxxx.

2. xxxxxx.

Etc.

Question descriptions:

The description for Question 1):

The overall objective(s) of the guideline is (are) specifically described.

1.1**Description**

The guideline should detail its objectives, including potential health impacts on society and the patient population. The benefits should be tailored to specific clinical issues and health projects. Examples of clear statements include:

- Preventing (long-term) complications in diabetes patients.
- Reducing the risk of vascular events in patients with a history of myocardial infarction.
- The most effective population-based colorectal disease screening strategy.
- Providing the most effective treatment and management guidelines for diabetes patients.

1.2**Locating Information**

© 2025 Wang B et al. *JAMA Network Open*.

Check the initial sections or paragraphs of the guideline for descriptions of its scope and purpose. Sometimes, the foundational theory and necessity of the guideline are in a separate document, such as a proposal. Typically, this information can be found under sections or chapters titled: Introduction, Scope, Purpose, Rules, Background, or Objectives.

1.3**How to Rate**

The content should include the following criteria:

- Health content (prevention, screening, diagnosis, treatment, etc.)
- Expected benefits or outcomes
- Target population (patients, societal groups)

[**Scoring Principle**: First, determine the presence or absence, then score based on the three criteria: Not mentioned (1 point); only the disease is described, without mentioning the population and expected outcomes (3-4 points); the disease and population are described, but not the expected outcomes (5-6 points); comprehensive description (7 points)]

The description for Question 2):

The health question(s) covered by the guideline is (are) specifically described.

2.1**Description**

The guideline should detail the health issues it addresses, even if not expressed in question form, particularly for key recommendations (see item 17 Key recommendations are easily identifiable). Continuing from the previous example:

- How many times a year should HbA1c be tested in diabetes patients?
- What daily dose of aspirin should be administered to confirmed acute myocardial infarction patients?
- Can population-based colorectal disease screening using fecal occult blood tests reduce colorectal cancer mortality?
- Is self-monitoring of blood glucose effective in controlling blood glucose in type 1 diabetes patients?

2.2**Locating Information**

Check the initial sections or paragraphs of the guideline for descriptions of its scope and purpose.

© 2025 Wang B et al. *JAMA Network Open*.

Sometimes, the foundational theory and necessity of the guideline are in a separate document, such as a retrieval strategy. Typically, this information can be found under sections or chapters titled: Introduction, Scope, Purpose, Rules, Background, or Objectives.

2.3**How to Rate**

The content should include the following criteria:

- Target population
- Interventions or exposure
- Comparators (if applicable)
- Outcomes
- Healthcare settings or environment

[**Scoring Principle**: First, determine the presence or absence, then score based on the four criteria: Not mentioned (1 point); only a rough description of related issues (3-4 points); detailed description of PIC, but lacking outcomes and settings (5 points); detailed description of PICOS (6-7 points)]

The description for Question 3):

The population (patients, public, etc.) to whom the guideline is meant to apply is specifically described.

3.1**Description**

There should be a clear description of the population covered by the guideline, including age range, gender, clinical type, and comorbidities. Examples include:

- The diabetes management guideline is limited to non-insulin-dependent diabetes patients without cardiovascular comorbidities.
- The depression management guideline is limited to severe depression classified by DSM-IV criteria, excluding psychotic symptoms and children.
- The breast cancer screening guideline is limited to women aged 50-70 without a cancer history and family history of breast cancer.

3.2**Locating Information**

Check the initial sections or paragraphs that describe the target population of the guideline.

© 2025 Wang B et al. *JAMA Network Open*.

Exclusion criteria (e.g., children) are also mentioned here. Typically, this information can be found under sections or chapters titled: Patient Population, Target Population, Relevant Patients, Scope, or Objectives.

3.3**How to Rate**

The content should include the following criteria:

- Target population, gender, age
- Clinical status (if relevant)
- Disease severity/stage (if relevant)
- Comorbidities (if relevant)
- Exclusion criteria (if relevant)

[**Scoring Principle**: First, determine the presence or absence, then score based on the five criteria: Not mentioned (1 point); rough description of population category (2-3 points); detailed description of population characteristics, including gender, age, clinical status (4-6 points); detailed description of population characteristics, providing detailed disease staging and exclusion criteria (7 points)]

The description for Question 4):

The guideline development group includes individuals from all relevant professional groups.

4.1**Description**

This item concerns the professionals involved in the guideline development process, including the initiation group, the research team selecting and evaluating evidence, and individuals forming the final recommendations, excluding those conducting external guideline evaluations (see item 13 The guideline has been externally reviewed by experts prior to its publication.) and target population representatives (see item 5 The views and preferences of the target population (patients, public, etc.) have been sought.). Information on the composition, principles, and relevant expertise of the guideline development group should be provided.

4.2**Locating Information**

Check the initial sections/paragraphs, acknowledgment sections, or appendices detailing the composition of the guideline development group. Typically, this information can be found under

© 2025 Wang B et al. *JAMA Network Open*.

sections or chapters titled: Methods, Guideline Group Members List, Acknowledgments, and Appendices.

4.3**How to Rate**

Each member of the guideline development group should include the following information:

- Name
- Discipline/Profession (e.g., Neurosurgeon, Methodologist)
- Institution (e.g., St. Peter's Hospital)
- Geographic Location (e.g., Seattle, Washington)
- Role in the guideline development group

[**Scoring Principle**: First, determine the presence or absence, then score based on the five criteria: Not mentioned (1 point); rough description of the group or names only (2-3 points); provided names, disciplines/professions (4 points); provided names, disciplines/professions, institutions (5 points); provided names, disciplines/professions, institutions, roles, geographic locations (6-7 points)]

The description for Question 5):

The views and preferences of the target population (patients, public, etc.) have been sought.

5.1**Description**

Clinical guidelines should consider the target population's experiences and consider the target population's views should be provided.

5.2**Locating Information**

Check the sections describing the guideline development process. Typically, this information can be found under sections or chapters titled: Scope, Methods, Guideline Group Members List, External Review, or Target Population Views.

5.3**How to Rate**

The content should include the following criteria:

- Statement on the methods used to obtain the views and preferences of patients/public (e.g., participation in the guideline development group, literature review)
- Statement on how the views and preferences were collected (e.g., literature evidence,

© 2025 Wang B et al. *JAMA Network Open*.

surveys, focus groups)

- Outcomes/Information obtained from patients/public
- Description of how the information was used in the guideline development process and/or forming recommendations

[**Scoring Principle**: First, determine the presence or absence, then score based on the four criteria: Not mentioned (1 point); considered patient preferences only (2 points); provided methods for collecting and obtaining patient preferences (3-4 points); provided collection methods, summarized, and used for guideline recommendations (5-7 points)]

The description for Question 6):

The target users of the guideline are clearly defined.

6.1**Description**

The guideline must specify its intended users to allow readers to quickly determine its relevance. For example, a guideline for lower back pain might include general practitioners, neurologists, orthopedic surgeons, rheumatologists, and physiotherapists.

6.2**Locating Information**

Check the initial sections or paragraphs describing the intended users of the guideline. Typically, this information can be found under sections or chapters titled: Users and Intended Users.

6.3**How to Rate**

The content should include the following criteria:

- Clear description of the intended readers of the guideline (e.g., specialists, family physicians, patients, clinical or institutional leaders/managers)
- Statement on how the intended users should use the guideline (e.g., informing clinical decisions, informing policies, informing care standards)

[**Scoring Principle**: First, determine the presence or absence, then score based on the two criteria: Not mentioned (1 point); vaguely mentioned the guideline users, such as specialists, family physicians, patients, clinical or institutional leaders/managers (2 points); clear statement of intended users (3-4 points); statement of users and how they should use the guideline, such as informing clinical decisions, policies, or care standards (6-7 points)]

© 2025 Wang B et al. *JAMA Network Open*.

Questions part:

Question 1):

The overall objective(s) of the guideline is (are) specifically described.

Question 2):
The health question(s) covered by the guideline is (are) specifically described.

Question 3):
The population (patients, public, etc.) to whom the guideline is meant to apply is specifically described.

Question 4):
The guideline development group includes individuals from all relevant professional groups.

Question 5):
The views and preferences of the target population (patients, public, etc.) have been sought.

Question 6):
The target users of the guideline are clearly defined.

【Second input】

Question descriptions:

The description for Question 7):

Systematic methods were used to search for evidence.

7.1.**Description**

Detailed search strategies for evidence should be provided, including search terms, information sources, and the time period covered by the literature. Information sources include electronic databases (e.g., MEDLINE, EMBASE, CINAHL), systematic review databases (e.g., Cochrane Library, DARE), manually searched journals, conference proceedings, and other guidelines (e.g., US National Guideline Clearinghouse, German Guidelines Clearinghouse). The search strategy should be understandable and eliminate bias, detailed enough for replication.

7.2**Locating Information**

Check the initial sections or paragraphs of the guideline development process. In some cases, the search strategy is described in a separate document or the guideline's appendix. Typically, this information can be found under sections or chapters titled: Methodology, Literature Search

© 2025 Wang B et al. *JAMA Network Open*.

Strategy, and Appendices.

7.3**How to Rate**

The content should include the following criteria:

- Search of known electronic databases or evidence repositories (e.g., MEDLINE, EMBASE, PsychINFO, CINAHL)
- Time span of the search (e.g., 2004-01-01 to 2008-03-31)
- Search terms used (e.g., subject terms, subterms, index terms)
- Complete search strategy (often in the appendix)

[**Scoring Principle**: First, determine the presence or absence, then score based on the four criteria: Not mentioned (1 point); mention of search databases without specific information (2 points); identification of search databases without specific search information (3 points); clear database and search terms (4 points); clear database, search time, search terms (5 points); clear database, search time, search terms, complete search strategy (6-7 points)]

The description for Question 8):

The criteria for selecting the evidence are clearly described.

8.1**Description**

Inclusion and exclusion criteria for evidence selection should be provided. These criteria and the reasons for excluding/including evidence should be clearly described. For example, the authors might decide to include only evidence from randomized controlled trials and exclude non-English literature.

8.2**Locating Information**

Check the initial sections or paragraphs of the guideline development process. In some cases, the criteria for including/excluding evidence are described in a separate document or appendix. Typically, this information can be found under sections or chapters titled: Methodology, Literature Search, Inclusion/Exclusion Criteria, Appendices.

8.3**How to Rate**

The content should include the following criteria:

- Description of inclusion criteria, including: target population (patients, public, etc.)

© 2025 Wang B et al. *JAMA Network Open*.

characteristics, study design, comparators (if relevant), outcomes, language (if relevant), setting (if relevant)

- Description of exclusion criteria (if relevant; e.g., inclusion criteria stating only French literature includes logically excludes non-French literature)

[**Scoring Principle**: First, determine the presence or absence, then score based on the two criteria: Not mentioned (1 point); mention of included study type only (2 points); mention of inclusion (or exclusion) criteria only (3-4 points); detailed inclusion and exclusion criteria (5-7 points)]

The description for Question 9):

The strengths and limitations of the body of evidence are clearly described.

9.1**Description**

The guideline should specify the strengths and limitations of the evidence, using formal or informal tools/methods to assess the risk of bias in individual studies and/or specific outcomes, and/or evaluate the overall body of evidence. This can be presented in various ways, such as tables comparing different quality domains, using formal tools or strategies (e.g., Jadad scale, GRADE method), or described in the text.

9.2**Locating Information**

Check the sections or chapters describing the guideline development process for information on study methodological quality (e.g., risk of bias). Evidence tables often summarize the characteristics of evidence quality. Some guidelines have distinct sections for describing and interpreting evidence.

9.3**How to Rate**

The content should include the following criteria:

Description of how to determine the presence of bias in the body of evidence and how the guideline development group interpreted these biases

Overall framework description, including: evidence body study design, methodological defects (sample size, blinding, allocation concealment, analysis methods), appropriateness/relevance of primary and secondary outcomes, consistency of all study results, direction of all study results,

© 2025 Wang B et al. *JAMA Network Open*.

benefit/harm balance, applicability to the practice context.

[**Scoring Principle**: First, determine the presence or absence, then score based on the criteria: Not mentioned (1 point); according to the content, the criteria are met, each criterion worth 1 point, e.g., only study design mentioned (2 points)]

The description for Question 10):

The methods for formulating the recommendations are clearly described.

10.1**Description**

The methods for formulating the recommendations and how final decisions were made should be described. Various methods can be used, such as voting, informal consensus, formal consensus meetings (e.g., Delphi method, Glaser method). The guideline should also specify any controversial areas and how disputes were resolved.

10.2**Locating Information**

Check the sections or chapters describing the guideline development process. In some cases, the methodology for formulating the final recommendations is in a separate document or appendix. Typically, this information can be found under sections or chapters titled: Methodology and Guideline Development Process.

10.3**How to Rate**

The content should include the following criteria:

- Description of the process for generating recommendations (e.g., steps in using the modified Delphi method, voting procedures)
- Results of the recommendation generation process (e.g., level of consensus reached using the modified Delphi method, voting results)
- Description of how the recommendation generation process affected the final recommendations (e.g., the impact of the Delphi method results on the final recommendations, consistency between final recommendations and voting results)

[**Scoring Principle**: First, determine the presence or absence, then score based on the three criteria: Not mentioned (1 point); provided recommendation generation method (2 points); provided recommendation generation method and described specific process/results, but no

© 2025 Wang B et al. *JAMA Network Open*.

method process (3-4 points); described generation process and provided results (5-6 points); comprehensive description of process, results, and impact on final recommendations (7 points)]

The description for Question 11):

The health benefits, side effects, and risks have been considered in formulating the recommendations.

11.1**Description**

When developing recommendations, guidelines should consider health benefits, adverse effects, and risks. For example, a breast cancer guideline might include discussions on the overall efficiency of various final outcomes, such as survival rates, quality of life, adverse reactions, symptom management, or comparing one treatment option with another. These issues should be presented as evidence.

11.2**Locating Information**

Check the sections or chapters describing the evidence body, interpreting and translating evidence into practical recommendations. Typically, this information can be found under sections or chapters titled: Methodology, Interpretation, Discussion, and Recommendations.

11.3**How to Rate**

The content should include the following criteria:

- Benefits reported and supporting data
- Harms/adverse reactions/risks reported and supporting data
- Reporting of the balance between benefits and harms/adverse reactions/risks
- Recommendations reflect consideration of both benefits and harms/adverse reactions/risks

[**Scoring Principle**: First, determine the presence or absence, then score based on the four criteria: Not mentioned (1 point); provided benefits or risks without supporting data (2 points); provided benefits and supporting data (3 points); provided benefits and risks with supporting data (4-5 points); consideration of benefit-harm balance (6-7 points)]

The description for Question 12):

There is an explicit link between the recommendations and the supporting evidence.

© 2025 Wang B et al. *JAMA Network Open*.

12.1**Description**

There should be a clear link between the guideline recommendations and the supporting evidence, allowing users to identify the evidence related to each recommendation.

12.2**Locating Information**

Identify and check the guideline recommendations and the background descriptions of the evidence supporting them. Typically, this information can be found under sections or chapters titled: Recommendations and Key Evidence.

12.3**How to Rate**

The content should include the following criteria:

- Description of how the guideline development group linked and used evidence to generate recommendations
- Each recommendation should be linked to key evidence descriptions/paragraphs and/or references
- Recommendations should be linked to evidence summaries/evidence tables in the guideline outcomes section

[**Scoring Principle**: First, determine the presence or absence, then score based on the three criteria: Not mentioned (1 point); description of how evidence led to recommendations/only references provided (2-3 points); description of how evidence led to recommendations and references provided (4-5 points); clear description of the relationship, references, and evidence summaries/tables (6-7 points). Note: If evidence is lacking or recommendations are consensus-based, clear statements or descriptions may be scored based on detail level (3-5 points)]

The description for Question 13):

The guideline has been externally reviewed by experts prior to its publication.

13.1**Description**

Guidelines should undergo external expert review before publication. Reviewers should not be members of the guideline development group and should include clinical experts, methodology experts, and possibly target population representatives (patients, public). The methodology for external review, including the reviewer list and their institutions, should be described.

© 2025 Wang B et al. *JAMA Network Open*.

13.2**Locating Information**

Check the sections or chapters describing the guideline development process and acknowledgments. Typically, this information can be found under sections or chapters titled: Outcomes, Interpretation, Acknowledgments.

13.3**How to Rate**

The content should include the following criteria:

- Purpose and intent of the external review (improving quality, collecting feedback on preliminary recommendations, evaluating feasibility and applicability, disseminating evidence)
- Methodology for ensuring external review (evaluation scale, open-ended inquiries)
- Description of the external review (e.g., number, type, institutions of reviewers)
- Outcomes/information collected from the external review (summary of significant findings)
- Description of how the information was used in reporting the guideline development process and/or forming recommendations (consideration of evaluation outcomes by the guideline group in forming final recommendations)

[**Scoring Principle**: First, determine the presence or absence, then score based on the criteria: Not mentioned (1 point); mention of guideline review without additional information (2-3 points); provided reviewer/group names (4 points); described review method/process/results, and reviewers/group (5 points); described review purpose, method/process/results, and reviewers/group names (6-7 points)]

The description for Question 14):

A procedure for updating the guideline is provided.

14.1**Description**

Guidelines should reflect the latest research findings, providing a clear statement on the update procedure. For example, providing a time interval or establishing a group that periodically reviews the literature and updates as required.

14.2**Locating Information**

Check the introductory sections describing the guideline development process and the final

© 2025 Wang B et al. *JAMA Network Open*.

sections. Typically, this information can be found under sections or chapters titled: Methodology, Guideline Update, Guideline Dates.

14.3**How to Rate**

The content should include the following criteria:

- Statement on guideline updates
- Clear time intervals or criteria for guiding recommendation formation

[**Scoring Principle**: First, determine the presence or absence, then score based on the criteria: Not mentioned (1 point); mention of updates without specific details (3 points); mention of update intervals or criteria (4-6 points); comprehensive description of update intervals and criteria (7 points)]

Questions part:

Question 7):
Systematic methods were used to search for evidence.

Question 8):
The criteria for selecting the evidence are clearly described.

Question 9):
The strengths and limitations of the body of evidence are clearly described.

Question 10):
The methods for formulating the recommendations are clearly described.

Question 11):
The health benefits, side effects, and risks have been considered in formulating the recommendations.

Question 12):
There is an explicit link between the recommendations and the supporting evidence.

Question 13):
The guideline has been externally reviewed by experts prior to its publication.

Question 14):
A procedure for updating the guideline is provided.

【Third input】

Question descriptions:

© 2025 Wang B et al. *JAMA Network Open*.

The description for Question 15):

The recommendations are specific and unambiguous.

15.1**Description**

The guideline should describe the recommendations precisely, specifying the circumstances and populations for which they apply, as described in the evidence body report.

- An example of a specific recommendation is: Children aged two or older diagnosed with acute otitis media should receive antibiotics if pain persists for more than three days or worsens after adequate analgesia at presentation. In some cases, a seven-day supply of amoxicillin will be given (dosage plan supply amount).
- An example of an ambiguous recommendation is: Antibiotics should be used in abnormal or complex cases. Sometimes, evidence is not always clear, and it can be challenging to determine the best approach. In such cases, it is important to highlight these uncertainties in the guideline.

15.2**Locating Information**

Identify and check the guideline recommendations. Typically, this information can be found under sections or chapters titled: Recommendations, Implementation Procedures Summary.

15.3**How to Rate**

The content should include the following criteria:

- Statement on the formation of recommendations
- Identification of the purpose or intent of the recommendations (improving quality of life, reducing adverse reactions)
- Identification of the relevant population (patients, public), and if applicable, statement on those not qualifying (e.g., recommendations not applicable to certain patients or conditions)

[**Scoring Principle**: First, determine the presence of the recommendations, then score based on the three criteria: vague and unclear if it is a recommendation (1 point); clearly identified as a recommendation, using terms like recommend, should, etc. (2-4 points); clearly identified as a recommendation and states purpose and/or population (5-7 points)]

The description for Question 16):

© 2025 Wang B et al. *JAMA Network Open*.

The different options for management of the condition or health issue are clearly presented.

16.1**Description**

A guideline aimed at managing a disease should consider various options for clinical screening, prevention, diagnosis, or treatment. These options should be explicitly presented. For example, recommendations for managing depression might include the following treatment options: a) TCA therapy b) SSRI therapy c) Psychotherapy d) Combined pharmacotherapy and psychotherapy

16.2**Locating Information**

Check the recommendations and their supporting evidence. Typically, this information can be found under sections or chapters titled: Implementation Summary, Recommendations, Discussion, Treatment Options, Treatment Alternatives.

16.3**How to Rate**

The content should include the following criteria:

- Description of options
- Description of the most suitable population or clinical status for each option

[**Scoring Principle**: First, determine the presence of options, then score based on the criteria: only one option mentioned (1-2 points); multiple options for the same issue (3-4 points); multiple options for the same issue with description of suitable populations or clinical status (5-7 points)]

The description for Question 17):

Key recommendations are easily identifiable.

17.1**Description**

Users should easily find the most relevant recommendations, addressing the main questions covered by the guideline. These recommendations should be identified in various ways, such as summarizing in a box, using bold text, underlining, flowcharts, or diagrams.

17.2**Locating Information**

Typically, this information can be found under sections or chapters titled: Implementation Summary, Conclusions, Recommendations. Some guidelines provide a separate summary for key recommendations (e.g., quick reference guides).

17.3**How to Rate**

© 2025 Wang B et al. *JAMA Network Open*.

- The content should include the following criteria:
- Recommendations are placed in a box, highlighted in bold text, underlined, or represented using flowcharts or diagrams.
- Specific recommendations are grouped and placed in a section.

[**Scoring Principle**: First, determine the presence of easily identifiable recommendations, then score based on the criteria: no easily identifiable recommendations (1 point); recommendations summarized in a box (or highlighted in bold text, underlined, or using flowcharts) (4-7 points)]

The description for Question 18):

The guideline describes facilitators and barriers to its application.

18.1**Description**

The application of the guideline may involve certain facilitators or barriers affecting the implementation of the recommendations. For example:

- A stroke guideline might recommend nursing care through stroke units and services, requiring a special funding mechanism to establish stroke units in the area.
- A primary care guideline for diabetes may require regular patient visits to diabetes clinics, prompting the establishment of such clinics in regions with a shortage of clinicians.

18.2**Locating Information**

Check the sections on guideline dissemination/implementation, or if possible, review additional documents with clear plans or strategies for guideline implementation. Typically, this information can be found under sections or chapters titled: Barriers, Guideline Application, Quality Indicators.

18.3**How to Rate**

- The content should include the following criteria:
- Identification of the types of facilitators and barriers
- Consideration of methods to collect facilitators and barriers (feedback from key participants, exploratory trials before widespread implementation)
- Information/description of facilitators and barriers types from surveys (e.g., practitioners' necessary skills to disseminate the recommendations, lack of equipment ensuring all eligible population receive mammography)

© 2025 Wang B et al. *JAMA Network Open*.

- Description of how this information affected the guideline development process and/or recommendation formation

[**Scoring Principle**: First, determine the presence of facilitators and barriers, then score based on the criteria: not mentioned (1 point); mention of facilitators or barriers (2-3 points); mention of both facilitators and barriers, with collection methods (4-5 points); mention of both facilitators and barriers, with collection or identification methods (6 points); description of factors and collection methods, and impact on recommendations (7 points)]

The description for Question 19):

The guideline provides advice and/or tools on how the recommendations can be put into practice.

19.1**Description**

To make a guideline more effective, additional materials may be needed to facilitate its implementation. These materials might include a summary, a quick reference guide, educational materials, results from exploratory trials, patient brochures, computer support, and any supplementary materials provided with the guideline.

19.2**Locating Information**

Check the sections on guideline dissemination/implementation. If available, specific supplementary materials will support the guideline's dissemination/implementation. Typically, this information can be found under sections or chapters titled: Tools, Resources, Implementation, Appendices.

19.3**How to Rate**

- The content should include the following criteria:
- The guideline includes an implementation section
- Tools and resources to facilitate guideline application: guideline outline documents, checklists, algorithms, connection with manuals, solutions for identified barriers (see item 18), tools for guideline promoters (see item 18), exploratory trials, and learning course results
- Guidance on how users can access tools and resources

[**Scoring Principle**: First, determine the presence of implementation tools, then score based on the criteria: not mentioned (1 point); implementation section or paragraph (2-4 points);

implementation section with tools/resources provided (5-6 points); implementation section, tools provided, and usage guidance (7 points)]

The description for Question 20):

The potential resource implications of applying the recommendations have been considered.

20.1**Description**

Recommendations may require additional resources, such as a more specialized team, new equipment, or expensive drug therapies. This may be related to healthcare budget costs, discussed in the guideline on the potential resource impact.

20.2**Locating Information**

Check the sections on guideline dissemination/implementation, or if possible, review additional documents with clear plans or strategies for guideline implementation. Some guidelines discuss related costs in sections on evidence or recommendation decisions. Typically, this information can be found under sections or chapters titled: Methodology, Cost-Use, Cost-Effectiveness, Collection Costs, Related Budget.

20.3**How to Rate**

The content should include the following criteria:

- Consideration of identifying cost information types (economic evaluation, drug acquisition costs)
- Methodology for collecting cost information (e.g., involvement of a health economist in the guideline development group, use of health technology assessment)
- Information/description of cost information from surveys (e.g., drug acquisition costs for each treatment course)
- Description of how the information was used in reporting the guideline development process and/or forming recommendations

[**Scoring Principle**: First, determine the presence of cost considerations, then score based on the criteria: not mentioned (1 point); mention of cost/economic issues/proposal for economic evaluation, etc. (3-4 points); specific survey information provided (5-6 points); survey information provided, and how it was used in guideline/recommendation formation (7 points)]

The description for Question 21):

The guideline presents monitoring and/or auditing criteria.

21.1**Description**

Measuring the application of guideline recommendations helps ensure their continued use, requiring clear standards derived from key recommendations. Standards can include process measures, behavioral measures, clinical or health outcomes measures. Examples of monitoring and auditing standards include:

- HbA1c should be less than 8.0%.
- Diastolic blood pressure should be less than 95mmHg.
- 80% of people over 50 should be screened for colorectal cancer using fecal occult blood tests.
- Amoxicillin should be used if acute otitis media symptoms persist for more than three days.

21.2**Locating Information**

Check the sections on guideline implementation/auditing, or if possible, additional documents with clear plans or strategies for guideline evaluation. Typically, this information can be found under sections or chapters titled: Recommendations, Quality Indicators, Auditing Standards.

21.3**How to Rate**

The content should include the following criteria:

- Identification of standards for implementing guidelines or complying with recommendations
- Identification of standards for evaluating the impact of implementing recommendations
- Information on the frequency and intervals of measurements
- Description or definition of how to measure outcomes

[**Scoring Principle**: First, determine the presence of monitoring/auditing criteria, then score based on the criteria: not mentioned (1 point); provision of indicators/evaluation standards (2-4 points); provision of indicators, and information on measurement frequency/intervals (5-6 points); provision of indicators, measurement frequency/intervals, and description of how to measure (7 points)]

The description for Question 22):

© 2025 Wang B et al. *JAMA Network Open*.

The views of the funding body have not influenced the content of the guideline.

22.1**Description**

Many guidelines use external sponsorship (e.g., government, professional bodies, charities, pharmaceutical companies). Support might come in the form of financial donations supporting the entire development or parts of it (e.g., printing the guideline). There should be a clear statement that the sponsor's views or interests did not influence the final recommendations.

22.2**Locating Information**

Check the sections describing the guideline development process or acknowledgments. Typically, this information can be found under sections or chapters titled: Declarations, Sponsorship Sources.

22.3**How to Rate**

The content should include the following criteria:

- Name of the sponsor or sponsorship source (or clear statement of no sponsorship)
- Statement that the sponsor did not influence the content of the guideline

[**Scoring Principle**: First, determine the presence of sponsorship, then score based on the criteria: not mentioned (1 point); provision of sponsorship source only (2 points); provision of sponsorship source and indication of influence (3 points); statement of no sponsorship (7 points); provision of sponsorship source and indication of no influence (7 points)]

The description for Question 23):

Competing interests of guideline development group members have been recorded and addressed.

23.1**Description**

Guideline development group members may have competing interests, such as financial ties to pharmaceutical companies. It must be clearly stated that all members have declared whether they have any competing interests.

23.2**Locating Information**

Check the sections describing the guideline development group or acknowledgments. Typically, this information can be found under sections or chapters titled: Methodology, Competing Interests, Guideline Group, Appendices.

23.3**How to Rate**

© 2025 Wang B et al. *JAMA Network Open*.

The content should include the following criteria:

- Description of the types of competing interests considered
- Methodology for collecting potential competing interests
- Description of competing interests
- Description of how competing interests influenced the guideline development process and recommendation formation

[**Scoring Principle**: First, determine the presence of competing interests, then score based on the criteria: not mentioned (1 point); mention of collection method/mention of competing interests/describing interest relationships without indicating conflict (3-4 points); mention of no competing interests without collection method (5 points); collection method for competing interests, report of competing interests, description of no influence (7 points); collection method for competing interests, report of competing interests, description of potential influence (6 points)]

Questions part:

Question 15):
The recommendations are specific and unambiguous.

Question 16):
The different options for management of the condition or health issue are clearly presented.

Question 17):
Key recommendations are easily identifiable.

Question 18):
The guideline describes facilitators and barriers to its application.

Question 19):
The guideline provides advice and/or tools on how the recommendations can be put into practice.

Question 20):
The potential resource implications of applying the recommendations have been considered.

Question 21):
The guideline presents monitoring and/or auditing criteria.

Question 22):
The views of the funding body have not influenced the content of the guideline.

© 2025 Wang B et al. *JAMA Network Open*.

Question 23):<br>Competing interests of guideline development group members have been recorded and addressed.

© 2025 Wang B et al. *JAMA Network Open*.

eAppendix 1. References of the 28 guidelines included

[1]. Higgins N, Tseng A, Sheehan NL, la Porte CJ. Antiretroviral therapeutic drug monitoring in Canada: current status and recommendations for clinical practice. Can J Hosp Pharm. 2009;62(6):500-509. doi:10.4212/cjhp.v62i6.849

[2]. Shipkova M, Hesselink DA, Holt DW, et al. Therapeutic Drug Monitoring of Everolimus: A Consensus Report. Ther Drug Monit. 2016;38(2):143-169. doi:10.1097/FTD.0000000000000260

[3]. Hiemke C, Bergemann N, Clement HW, et al. Consensus Guidelines for Therapeutic Drug Monitoring in Neuropsychopharmacology: Update 2017. Pharmacopsychiatry. 2018;51(1-02):9-62. doi:10.1055/s-0043-116492

[4]. Rybak MJ, Lomaestro BM, Rotschafer JC, et al. Vancomycin therapeutic guidelines: a summary of consensus recommendations from the infectious diseases Society of America, the American Society of Health-System Pharmacists, and the Society of Infectious Diseases Pharmacists. Clin Infect Dis. 2009;49(3):325-327. doi:10.1086/600877

[5]. Aonuma K, Shiga T, Atarashi H, et al. Guidelines for Therapeutic Drug Monitoring of Cardiovascular Drugs Clinical Use of Blood Drug Concentration Monitoring (JCS 2015) - Digest Version. Circ J. 2017;81(4):581-612. doi:10.1253/circj.CJ-66-0138

[6]. Acosta EP, Gerber JG; Adult Pharmacology Committee of the AIDS Clinical Trials Group. Position paper on therapeutic drug monitoring of antiretroviral agents. AIDS Res Hum Retroviruses. 2002;18(12):825-834. doi:10.1089/08892220260190290

[7]. Argoff C E, Alford D P, Fudin J, et al. Rational urine drug monitoring in patients receiving opioids for chronic pain: consensus recommendations. Pain Medicine, 2018, 19(1): 97-117.

[8]. Hammett-Stabler CA, Johns T. Laboratory guidelines for monitoring of antimicrobial drugs. National Academy of Clinical Biochemistry. Clin Chem. 1998;44(5):1129-1140.

[9]. Matsumoto K, Takesue Y, Ohmagari N, et al. Practice guidelines for therapeutic drug monitoring of vancomycin: a consensus review of the Japanese Society of Chemotherapy and the Japanese Society of Therapeutic Drug Monitoring. J Infect Chemother. 2013;19(3):365-380. doi:10.1007/s10156-013-0599-4

[10]. Okada K, Kimura T, Mikamo H, et al. Clinical practice guidelines for therapeutic drug monitoring of arbekacin: a consensus review of the Japanese Society of Chemotherapy and the Japanese Society of Therapeutic Drug Monitoring. J Infect Chemother. 2014;20(1):1-5. doi:10.1016/j.jiac.2013.08.008

[11]. He Z, Qiu K.Vancomycin individualized administration guidelines for clinical pharmacists. Pharm Today. 2015; 2015:78-82

[12]. Ye ZK, Chen YL, Chen K, et al. Therapeutic drug monitoring of vancomycin: a guideline of the Division of Therapeutic Drug Monitoring, Chinese Pharmacological Society. J Antimicrob Chemother. 2016;71(11):3020-3025. doi:10.1093/jac/dkw254

[13]. Ashbee HR, Barnes RA, Johnson EM, Richardson MD, Gorton R, Hope WW. Therapeutic drug monitoring (TDM) of antifungal agents: guidelines from the British Society for Medical Mycology. J Antimicrob Chemother. 2014;69(5):1162-1176. doi:10.1093/jac/dkt508

[14]. Hamada Y, Tokimatsu I, Mikamo H, et al. Practice guidelines for therapeutic drug monitoring of voriconazole: a consensus review of the Japanese Society of Chemotherapy and the Japanese Society of Therapeutic Drug Monitoring. J Infect Chemother. 2013;19(3):381-392. doi:10.1007/s10156-013-0607-8

© 2025 Wang B et al. *JAMA Network Open*.

[15]. Chau M M, Kong D C M, Van Hal S J, et al. Consensus guidelines for optimising antifungal drug delivery and monitoring to avoid toxicity and improve outcomes in patients with haematological malignancy, 2014. Internal medicine journal, 2014, 44(12b): 1364-1388.
[16]. Chinese Pharmacological Society. Individualized medication of voriconazole: a practice guideline of the division of therapeutic drug monitoring Chinese Pharmacological Society. 2017 Available at. http://www.tdmchina.org/portal.php?mod=view&aid=467.
[17]. Verheijen RB, Yu H, Schellens JHM, Beijnen JH, Steeghs N, Huitema ADR. Practical Recommendations for Therapeutic Drug Monitoring of Kinase Inhibitors in Oncology. Clin Pharmacol Ther. 2017;102(5):765-776. doi:10.1002/cpt.787
[18]. Oellerich M, Armstrong VW, Schütz E, Shaw LM. Therapeutic drug monitoring of cyclosporine and tacrolimus. Update on Lake Louise Consensus Conference on cyclosporin and tacrolimus. Clin Biochem. 1998;31(5):309-316. doi:10.1016/s0009-9120(98)00049-6
[19]. Yatscoff RW, Boeckx R, Holt DW, et al. Consensus guidelines for therapeutic drug monitoring of rapamycin: report of the consensus panel. Ther Drug Monit. 1995;17(6):676-680. doi:10.1097/00007691-199512000-00022
[20]. Holt DW, Armstrong VW, Griesmacher A, et al. International Federation of Clinical Chemistry/International Association of Therapeutic Drug Monitoring and Clinical Toxicology working group on immunosuppressive drug monitoring. Ther Drug Monit. 2002;24(1):59-67. doi:10.1097/00007691-200202000-00011
[21]. Morris RG, Ilett KF, Tett SE, et al. Cyclosporin monitoring in Australasia: 2002 update of consensus guidelines. Ther Drug Monit. 2002;24(6):677-688. doi:10.1097/00007691-200212000-00001
[22]. Cantarovich M, Brown N W, Ensom M H H, et al. Mycophenolate monitoring in liver, thoracic, pancreas, and small bowel transplantation: a consensus report. Transplantation Reviews, 2011, 25(2): 65-77.
[23]. Trevillian P; Caring for Australians with Renal Impairment (CARI). The CARI guidelines. Calcineurin inhibitors in renal transplantation: therapeutic drug monitoring. Nephrology (Carlton). 2007;12 Suppl 1:S57-S65. doi:10.1111/j.1440-1797.2007.00730.x
[24]. The National Institute for Health and Care Excellence (NICE). Therapeutic monitoring of TNF-alpha inhibitors in Crohn's disease (LISATRACKER ELISA kits, IDKmonitor ELISA kits, and Promonitor ELISA kits).Available at: https://www.nice.org.uk/guidance/dg22. Date: 2016
[25]. Mitrev, N.Therapeutic drug monitoring guided anti-tumour necrosis factor therapy in inflammatory bowel disease (IBD): Gastroenterological Society of Australia (GESA)/Australian IBD Assoc (aibda) consensus statements. Available at: https://ses.library.usyd.edu.au/bitstream/2123/17449/2/Mitrev_N_thesis.pdf. Date: 2017
[26]. Patsalos PN, Berry DJ, Bourgeois BF, et al. Antiepileptic drugs--best practice guidelines for therapeutic drug monitoring: a position paper by the subcommission on therapeutic drug monitoring, ILAE Commission on Therapeutic Strategies. Epilepsia. 2008;49(7):1239-1276. doi:10.1111/j.1528-1167.2008.01561.x
[27]. Dreesen E, Bossuyt P, Mulleman D, Gils A, Pascual-Salcedo D. Practical recommendations for the use of therapeutic drug monitoring of biopharmaceuticals in inflammatory diseases. Clin Pharmacol. 2017;9:101-111. Published 2017 Oct 3. doi:10.2147/CPAA.S138414
[28]. Feuerstein JD, Nguyen GC, Kupfer SS, Falck-Ytter Y, Singh S; American

© 2025 Wang B et al. *JAMA Network Open*.

Gastroenterological Association Institute Clinical Guidelines Committee. American Gastroenterological Association Institute Guideline on Therapeutic Drug Monitoring in Inflammatory Bowel Disease. Gastroenterology. 2017;153(3):827-834.

© 2025 Wang B et al. *JAMA Network Open*.
