# Supplementary material for: Evaluating the Quality of Guidelines Using the AGREE II Tool by a Large Language Model vs Human Appraisers
Source: JAMA Netw Open. 2025 May 28;8(5):e2512621. doi: 10.1001/jamanetworkopen.2025.12621 (PMC12120650; doi:10.1001/jamanetworkopen.2025.12621)
Supplement: Supplement 2. — Data Sharing Statement [file jamanetwopen-e2512621-s002.pdf]

Data Sharing Statement

Wang. Evaluating the Quality of Guidelines Using the AGREE II Tool by a Large Language Model vs Human Appraisers. *JAMA Netw Open*. Published May 28, 2025. doi:10.1001/jamanetworkopen.2025.12621

Data
Data available: Yes
Data types: Data (not involving human participants)
How to access data: wangbingyi@chevidence.cn
When available: With publication

Supporting Documents
Document types: None

Additional Information
Who can access the data: The data will be made available to researchers for purposes of reproducing the results or conducting secondary analyses. Requests for data access should be directed to the corresponding authors (chevidence@lzu.edu.cn).
Types of analyses: This data is used to assist clinical workers or researchers in quickly evaluating the quality of guidelines
Mechanisms of data availability: With investigator support
